# Using Mesenchymal Stem Cells to Treat Female Infertility: An Update on Female Reproductive Diseases

**DOI:** 10.1155/2019/9071720

**Published:** 2019-12-06

**Authors:** Yun-xia Zhao, Shao-rong Chen, Ping-ping Su, Feng-huang Huang, Yan-chuan Shi, Qi-yang Shi, Shu Lin

**Affiliations:** ^1^Department of Gynaecology and Obstetrics, The Second Affiliated Hospital of Fujian Medical University, Quanzhou, Fujian Province, China; ^2^Diabetes and Metabolism Division, Garvan Institute of Medical Research, 384 Victoria Street, Darlinghurst, Sydney, NSW 2010, Australia; ^3^Faculty of Medicine, St. Vincent's Clinical School, UNSW Sydney, NSW 2052, Australia; ^4^Department of Cardiology, Southwest Hospital, Third Military Medical University (Army Medical University), China

## Abstract

Female infertility impacts the quality of life and well-being of affected individuals and couples. Female reproductive diseases, such as primary ovarian insufficiency, polycystic ovary syndrome, endometriosis, fallopian tube obstruction, and Asherman syndrome, can induce infertility. In recent years, translational medicine has developed rapidly, and clinical researchers are focusing on the treatment of female infertility using novel approaches. Owing to the advantages of convenient samples, abundant sources, and avoidable ethical issues, mesenchymal stem cells (MSCs) can be applied widely in the clinic. This paper reviews recent advances in using four types of MSCs, bone marrow stromal cells, adipose-derived stem cells, menstrual blood mesenchymal stem cells, and umbilical cord mesenchymal stem cells. Each of these have been used for the treatment of ovarian and uterine diseases, and provide new approaches for the treatment of female infertility.

## 1. Introduction

Infertility is defined as the failure to achieve any pregnancy (including a miscarriage) for at least 12 months [1]. In 2002, 7.4% of married women, or about 2.1 million women, were infertile in the United States [2]. The causes of infertility can be divided into three main categories for which the prevalence is variable: female causes (33 to 41%), male causes (25 to 39%), and mixed causes (9 to 39%) [3]. These statistics highlight the impressive numbers of women undergoing infertility.

There are many factors causing female infertility, among which reproductive system-related diseases are the main causes. Etiologies for female infertility include ovulation disorders (polycystic ovary syndrome, hypothalamic dysfunction, and primary ovarian insufficiency), tubal infertility, endometriosis, and uterine and cervical causes (cervical stenosis, polyps, and tumors). Hormone replacement therapy is effective in some types of infertility, but there is substantial evidence from observational studies that such therapy increases the risk of breast cancer [4, 5]. Ovulation induction, superovulation, or assisted reproductive technologies have shown trends toward increased pregnancy rates, though different factors relating to the increased risks for multiple pregnancies must be considered [6]. These findings indicate shortcomings of existing treatment regimens.

Scientists have investigated other therapeutic measures, such as stem cell therapy, for infertility. Stem cells are undifferentiated cells with the ability to renew themselves for long periods without significant changes in their general properties. They can differentiate into various specialized cell types under certain physiological or experimental conditions. Due to the limitations of using embryonic and induced pluripotent stem cells in the clinic, there is great interest in mesenchymal stem cells (MSCs), which are free of both ethical concerns and teratoma formation [7].

MSCs, also called mesenchymal stromal cells, are a subset of nonhematopoietic adult stem cells that originate from the mesoderm. They possess self-renewal abilities and multilineage differentiation into not only mesoderm lineages, such as chondrocytes, osteocytes, and adipocytes, but also ectodermic and endodermic cells [8–10]. MSCs can be harvested from several adult tissues, such as bone marrow, menstrual blood, adipose tissue, the umbilical cord, and placenta [11–15].

## 2. Causes of Infertility in Female Reproductive Organs

**Figure 1:**
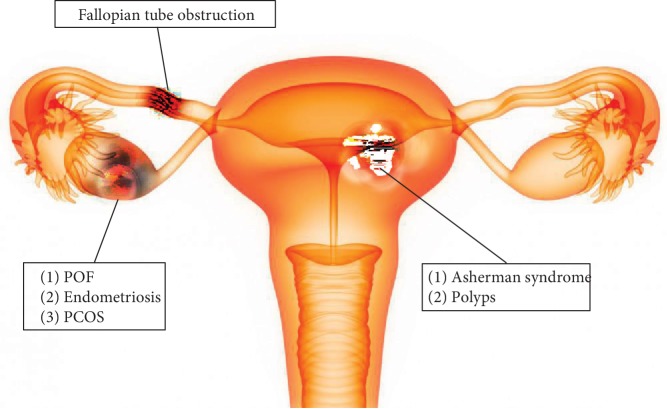
Diagram showing some possible causes of female infertility, such as fallopian tube obstruction, premature ovarian failure (POF), endometriosis, polycystic ovary syndrome (PCOS), Asherman syndrome, and polyps.

Causes of infertility in female reproductive organs include premature ovarian failure (POF), polycystic ovary syndrome, endometriosis, fallopian tube obstruction, Asherman syndrome, and other, less frequent anomalies of the reproductive tract (Figure 1, Table 1).

## 3. Mesenchymal Stem Cells

**Figure 2:**
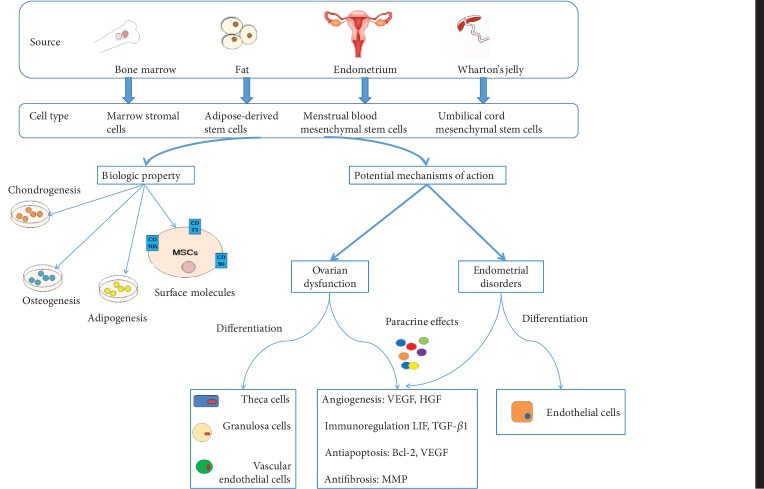
The derivation of the four types of MSCs and the biologic property of these MSCs. Potential mechanisms have been proposed for ovarian dysfunction and endometrial disorder therapy. Vascular endothelial growth factor (VEGF), hepatocyte growth factor (HGF), leukemia inhibitory factor (LIF), transforming growth factor (TGF), B-cell lymphoma 2 (Bcl-2), and matrix metalloproteinase (MMP).

To begin to address the use of mesenchymal stem cells (MSCs), the Mesenchymal and Tissue Stem Cell Committee of the International Society for Cellular Therapy has proposed minimal criteria to define human MSCs. First, MSCs must be plastic-adherent when maintained in standard culture conditions. Second, MSCs must express CD105, CD73, and CD90, and lack expression of CD45, CD34, CD14 or CD11b, CD79a or CD19, and HLA-DR surface molecules. Third, MSCs must differentiate to osteoblasts, adipocytes, and chondroblasts in vitro [8]. In 2016, the institute recommended adding MSC immunomodulation function-related factor detection as a supplementary test standard [16, 17]. The different MSCs are classified based on their source (Figure 2).

Laboratory experiments and clinical trials are now using MSCs, alone or in combination with other drugs, for potential application to ovarian dysfunction and endometrial disorders (Table 2) [18–21]. Importantly, therapeutic interventions for numerous diseases in female reproductive organs are causing great excitement. More importantly, these studies provide a desirable experimental model for elucidating the underlying mechanism of using MSCs for treating female infertility. This provides the theoretical basis for further studies and clinical therapy with MSCs.

For ovarian dysfunction, MSCs can directly and impulsively migrate to the injured ovary and survive there under the stimulation of multiple factors, which facilitates ovarian recovery. According to available studies, the number of differentiated and functionally integrated MSCs is too small to explain the observed improvements in ovarian function. Furthermore, whether MSCs differentiate into oocytes after migrating to injured tissue is still unknown. Improved ovarian function in these studies might be driven by paracrine mechanisms. These mechanisms involve the secretion of certain cytokines, including vascular endothelial growth factor, insulin-like growth factor, and hepatocyte growth factor, which are helpful for angiogenesis, anti-inflammation, immunoregulation, antiapoptosis, and antifibrosis to help ovarian restoration.

Further studies are needed to explore whether MSCs differentiate into target cells such as oocytes or supporting cells that improve ovarian functions and ultimately correct ovarian dysfunction. Such differentiation would also be valuable for MSC transplantation applied as a clinical therapy. Similarly, MSCs improve the endometrial reserve, which mainly depends on homing and paracrine activities. In studies to date, it is widely accepted that the paracrine effect of MSCs is the most important, rather than differentiation. Specifically, the regenerative properties of transplanted MSCs can be attributed to mechanisms that involve cell-cell contact and secretion of bioactive molecules that promote angiogenesis and tissue repair, thereby inhibiting scarring, modulating inflammatory and immune reactions, and activating tissue-specific progenitor cells. However, other research suggests that MSCs engraft the endometrium in rodents and humans, where they become epithelial, stromal, and endothelial cells. Thus, MSCs might promote endometrial regeneration and restore fertility by paracrine factors, but other mechanisms are plausible.

### 3.1. Bone Marrow Stromal Cells

Initially described by Owen and Friedenstein in 1988 [22], bone marrow stromal cells were separated from nucleated bone marrow cells on plastic culture dishes by density gradient centrifugation [23]. These cells had a longer replication cycle and premature senility, accounting for only 0.01–0.001% of nucleated bone marrow cells [24]. Bone marrow stromal cells, which have been the main source of multipotent stem cells, serve as a standard for comparison with MSCs from other sources [25]. Bone marrow stromal cells not only commit to osteoblasts, adipocytes, and chondroblasts, but also differentiate into granulosa [26], endometrial [27, 28], and endothelial cells [29] in mammals. Furthermore, bone marrow stromal cells have broad application prospects in the field of regenerative medicine, including reproductive dysfunction [30].

#### 3.1.1. Application of Bone Marrow Stromal Cells to Treat Ovarian Dysfunction

Several studies have shown beneficial effects of bone marrow stromal cell treatment in a chemotherapy-induced ovarian failure animal model. Specifically, the results showed that ovarian structure and functions could be restored by bone marrow stromal cells [31–34]. Although chemotherapy drugs can inhibit the growth of tumor cells, they can also lead to granulosa cell apoptosis, follicular atresia, ovarian function decline, and other manifestations of premature ovarian failure. Granulosa cells, which are located on the lateral side of the oocyte zona pellucidum and secrete estrogen under the action of follicle-stimulating hormone and other paracrine factors, play a role in nutrition and support of oocytes. Granulosa cell apoptosis thus leads to a decrease in estrogen levels in the body, affecting the normal development of oocytes.

In 2013, Abd-Allah et al. used bone marrow stromal cells from male rabbits to treat cyclophosphamide-induced ovarian failure and discovered that the ovarian functional reserve and number of follicles were improved. In addition, there were increased estrogen and vascular endothelial growth factor levels, reduced follicle-stimulating hormone levels, and diminished caspase-3 expression [31]. Badawy et al., in 2017, showed that bone marrow stromal cells were able to restore ovaries damaged by chemotherapy in mice. Furthermore, the animals regained their fertility [32]. Another study found that bone marrow stromal cells overexpressed miR-21, the earliest discovered microRNA, and this microRNA improved chemotherapy-induced POF in rats, possibly by downregulating phosphatase and tensin homolog and programmed cell death protein 4 to decrease granulosa cell apoptosis [33].

#### 3.1.2. Application of Bone Marrow Stromal Cells to Treat Endometrial Disorders

The uterine endometrium is a dynamic tissue that consists of a glandular epithelium and stroma that undergoes regeneration in each reproductive cycle. The uterine endometrium can be structurally divided into two zones: the upper functional layer and lower basal layer, which regenerates a new functional layer according to fluctuating levels of estrogen and progesterone. In humans, bone marrow stromal cells identified in the uterine endometrium participate in the regeneration of endometrial tissue [35, 36]. Implantation of autologous bone marrow stromal cells to treat endometrial injury restored menstruation in five out of six cases [37]. In animal models, bone marrow stromal cells have been used successfully to treat thin endometrium by locating them in this tissue where they differentiated into numerous cells and exerted immunomodulatory effects [27]. Additionally, bone marrow stromal cells restored functional endometrium in patients with Asherman syndrome and improved the reproductive outcomes [27, 38, 39].

In both preclinical animal models and human clinical trials, CD133^+^ bone marrow stromal cells induce endometrial proliferation by engrafting around endometrial vessels of the traumatized endometrium and secreting specific growth factors, such as thrombospondin-1 and insulin-like growth factor-1 [40, 41]. To better compensate for the insufficient intrinsic regeneration ability of the endometrium, inhibit fibrosis, promote angiogenesis, and improve endometrial receptivity, collagen scaffolds with bone marrow stromal cells have been introduced into treatments for Asherman syndrome [42].

### 3.2. Adipose-Derived Stem Cells

Currently, adipose-derived stem cells, a new type of MSC, have been used primarily to repair tissues [43–45]. Although these cells have the same biologic characteristics as bone marrow stromal cells [46], they are easier to isolate in large quantities (by minimally invasive liposuction) than bone marrow stromal cells [47]. Thus, compared with bone marrow stromal cells, adipose-derived stem cells represent a more practical option.

Damous et al. demonstrated that adipose-derived stem cell therapy improved ovarian graft quality by promoting an increase in vascular endothelial growth factor-A gene expression and the number of blood vessels in ovarian tissue to induce an earlier resumption of function in freshly grafted ovaries of adult female rats [48]. In addition, adipose-derived stem cells ameliorated chemotherapy-induced ovarian dysfunction in mouse models and were capable of inducing angiogenesis and restoring the number of ovarian follicles and corpus luteum in damaged ovaries [49, 50]. Another experiment, using a rat model of premature ovarian insufficiency, verified that adding a collagen scaffold enhanced the short-term maintenance of adipose-derived stem cells in ovaries, compared with transplanting these cells alone [51]. In another experimental rat model, the use of estrogen in combination with adipose-derived stem cells efficiently induced regeneration of the endometrium in Asherman syndrome therapy [52].

### 3.3. Menstrual Blood Mesenchymal Stem Cells (MB-MSCs)

Menstrual blood mesenchymal stem cells (MB-MSCs) can be isolated from menstrual blood [12]. These cells have high proliferative, self-renewal, and multiple differentiation potentials. In addition, they appear to possess numerous advantages over stem cells derived from other sources including ease of collection, safe and noninvasive proliferation, no ethical concerns, and no autoimmune rejection responses [12, 53]. Some clinical trials have used MB-MSCs to treat neuronal diseases [54, 55], diabetes mellitus [56, 57], and multiple sclerosis [53].

#### 3.3.1. Application of MB-MSCs to Treat Ovarian Dysfunction

Several studies have shown that MB-MSCs reduce apoptosis in granulosa cells and fibrosis of the ovarian interstitium, thereby improving folliculogenesis and rescuing overall ovarian function in an animal model of POF [58, 59], including restoring fertility [60]. In addition, Wang et al. demonstrated that MB-MSCs produced a high level of fibroblast growth factor 2, which enhanced cell survival, proliferation, and function to repair tissue damage [61, 62]. Furthermore, Yan et al. indicated that MB-MSCs reduced granulosa cell apoptosis and improved ovarian functions in mice by downregulating Gadd45b protein expression (a stress sensor whose effects are mediated via physical interactions with other cellular proteins implicated in cell cycle regulation) and upregulating cyclinB1 and CDC2 (regulators of the G2/M transition in mammalian cells) [63–66].

#### 3.3.2. Application of MB-MSCs to Treat Endometrial Disorders

MB-MSCs isolated from ectopic endometriotic lesions contribute to the pathogenesis of endometriosis [67, 68]. A clinical study where autologous MB-MSCs were transplanted into seven patients with severe Asherman syndrome, followed by hormonal stimulation, showed that the thickness of the endometrium in five women reached 7 mm, one patient had a spontaneous pregnancy, and two of the remaining four patients undergoing embryo transfer became pregnant [69].

In rats with damaged endometrium (an Asherman syndrome model), transplanted MB-MSCs assembled into spheroids and significantly improved fertility by increasing the synthesis of angiogenic and anti-inflammatory factors [70]. The main properties of MB-MSCs were retained in the spheroids, except for the expression of CD146 that was negatively correlated with self-renewal ability [71]. This seems to be a key to improve the therapeutic effect of MB-MSCs organized into spheroids.

Zheng et al. were the first to show that MB-MSCs could differentiate into endometrial cells in vitro and rebuild endometrial tissue in NOD-SCID mice after administering estrogen and progesterone in vivo [72]. As a transcription factor, OCT-4-positive cells can differentiate into three germ layers [73]. Furthermore, the cloning efficiency and OCT-4 expression of MB-MSCs from patients with severe intrauterine adhesions were significantly decreased compared with controls [72].

Platelet-rich plasma (PRP), an autologous plasma product with platelet concentrations above baseline values, has been used to treat acute and chronic injuries [74, 75]. Zhang et al. compared placebo, MB-MSC transplantation, PRP transplantation, and combined MB-MSC and PRP transplantation in the treatment of a rat model of intrauterine adhesion [76]. They found that combining MB-MSCs with PRP was more effective than either treatment alone in improving endometrial proliferation, angiogenesis, and morphological recovery. This treatment also reduced fibrosis and inflammation by changing the Hippo signaling pathway and regulating the downstream factors, connective tissue growth factor, Wnt5a, and Gdf5.

### 3.4. Umbilical Cord Mesenchymal Stem Cells

Umbilical cord mesenchymal stem cells (UC-MSCs), isolated directly from Wharton's jelly of the UC, are called human Wharton's jelly MSCs. They express the MSC markers CD29, CD44, CD73, CD90, and CD105, and do not express CD31, CD45, and HLA-DR85. Because they have lower oncogenicity and faster self-renewal abilities compared to other sources of MSCs, UC-MSCs are a new source of stem cells that can differentiate into several mesodermal cell types and be used for cell therapy.

#### 3.4.1. Application of UC-MSCs to Treat Ovarian Dysfunction

UC-MSCs have been used in several animal models to successfully treat POF by reducing apoptosis of granulosa cells, decreasing follicle-stimulating hormone serum levels, and increasing estrogen and anti-Mullerian hormone levels [77–80]. Elfayomy et al. proposed that UC-MSCs could reverse paclitaxel-induced apoptosis of ovarian cells either by establishing a normal arrangement of the surface epithelium and tunica albuginea, or by upregulating cytokeratin 8/18, transforming growth factor-*β*, and proliferating cell nuclear antigen to suppress caspase-3 expression [81]. In another investigation, Jalalie et al. transplanted CM-Dil-labeled human UC-MSCs into cyclophosphamide-injured ovaries in mice [82]. They found that UC-MSCs were not distributed equally in different parts of the ovarian tissue. Specifically, the number of CM-Dil-labeled human UC-MSCs in the ovarian medulla was greater than those of the ovarian cortex and germinal epithelium.

UC-MSCs on a collagen scaffold have been transplanted into ovaries to treat POF [83, 84]. Ding et al. found that this technique activated primordial follicles in vitro via phosphorylation of FOXO3a, a major suppressor of primordial follicle activation, and FOXO1. Li et al. found that human UC-MSCs used to treat perimenopausal rats secreted cytokines, such as hepatocyte growth factor, vascular endothelial growth factor, and insulin-like growth factor-1, resulting in improved ovarian reserve functions [85].

#### 3.4.2. Application of UC-MSCs to Treat Endometrial Disorders

Wharton's jelly-derived MSCs have the ability to differentiate into endometrial cells [86]. In a rat model, Zhang et al. found that human UC-MSCs repaired injured endometrium, thereby improving fertility. These researchers also found that the number of implanted embryos was higher in groups with multiple UC-MSC transplantations compared to a single UC-MSC transplantation, by upregulating vascular and downregulating proinflammatory factors [87]. Furthermore, UC-MSCs in collagen scaffolds have been used to promote endometrial regeneration by upregulating matrix metalloproteinase-9 in rat uterine scars [88, 89].

UC-MSCs can ameliorate damage to human endometrial stromal cells [90], and local intramuscular injection is effective for treating uterine niches after cesarean delivery [91]. Additionally, UC-MSCs on collagen scaffolds have been used in a phase I clinical trial to treat patients with recurrent uterine adhesions. The results suggested that they can improve endometrial proliferation, differentiation, and neovascularization by upregulating estrogen receptor *α*, vimentin, Ki67, and von Willebrand factor expression levels, and downregulating the ΔNP63 expression level [92].

## 4. Conclusions and Future Perspectives

MSCs have demonstrated great potential and availability for treating female infertility in animal and human studies. Autologous adipose-derived stem cells are especially useful because they are not only easily obtained, but also avoid graft rejection after transplantation. In recent decades, autologous adipose-derived stem cell transplantation or injection have shown positive effects on rat models of POF and Asherman syndrome and can increase fertilization rates. However, there are several main directions for using MSC to treat infertile women caused by ovarian or uterine factors: (1) Most studies have been done on small animals, and there is a serious lack of valuable research in large animal models that more closely mimic the ovarian or endometrial pathophysiology of human female infertility. Furthermore, a randomized controlled trial should be conducted to confirm the therapeutic effect of MSCs in fertility medicine. (2) The mechanism of MSCs in treating dysfunction of female reproductive organs is still unknown. Possibilities include promoting angiogenesis, differentiating into functional cells, and a paracrine mechanism. Among these, a paracrine mechanism might be the most important for female infertility treatment. However, beneficial paracrine factors remain unknown and multiple mechanisms may be synergistic. (3) While MSC therapy is promising, the limited survival and engraftment of bioactive agents due to a hostile environment is a bottleneck for disease treatment. Therefore, how to maintain and enhance the survival and secretion of MSCs over a longer period of time requires more in-depth research. One approach that maximizes the utility of MSCs for ovarian and endometrial disorders has been the development of various types of biomaterials. Collagen-based biomaterials have already been used as MSC delivery vehicles to enhance cell adhesion, retention, and engraftment. Nevertheless, additional work is needed to optimize this approach.

## Figures and Tables

**Table 1:** Causes of infertility in female reproductive organs.

Disease	Etiologies	Definition
POF	Genetic defects, autoimmune processes, chemotherapy, radiation, and infections	Cessation of ovarian function after menarche but before the age of 40, without or with ovarian follicle depletion

PCOS	Maternal PCOS, intrauterine hyperandrogenism, inflammatory adipokines, aboriginal origin-Western diet	A complex disorder characterized by infertility, hirsutism, obesity, and various menstrual disturbances

Endometriosis	Oxidative stress, reactive oxygen species, antioxidants and inflammatory, genetic, and epigenetic factors	A condition in which functional endometrial tissue is present outside the uterus

Fallopian tube obstruction	Neoplasms, neoplasms, tuboovarian abscess	Tubal obstruction is caused by inflammation of the fallopian tube or pelvic peritoneum

AS	Trauma, infection, low level of estradiol, repeated or aggressive curettage, severe endometritis	Absence of a normal opening in the lumen of the female genital tract, from the fallopian tubes to the vagina

POF: premature ovarian failure; PCOS: polycystic ovary syndrome; AS: Asherman syndrome.

**Table 2:** Application of MSC therapy in the treatment of female reproductive dysfunction.

MSC types	Disease		Treatment	Model	Main results	References
Bone marrow stromal cells	Ovarian dysfunction	CTX-induced ovarian failure	Intravenous injection	Rabbit	Ovarian function ↑	31
		CTX-induced ovarian failure	Local injection	Mice	Restore ovarian hormone production	34
	Endometrial disorders	24-gauge needle-induced AS	Labeled with SPIOs local/tail vein injection	Mice	Endometrial proliferation ↑	41
		Refractory AS	Uterine artery injection	Human	Reconstruct the endometrium	40

Adipose-derived stem cells	Ovarian dysfunction	Cisplatin-induced ovarian failure	Local injection	Mice	Ovarian function ↑	49
		TG-induced ovarian damage	Collagen scaffold	Rat	Fertility ↑	51
	Endometrial disorders	Trichloroacetic acid-induced AS	Intraperitoneal injection	Rat	Fibrosis ↓, endometrial proliferation ↑	52

MB-MSCs	Ovarian dysfunction	CTX-induced POF	Local injection	Mice	Ovarian weight ↑, hormone secretion ↓	59
		Cisplatin-induced POF	Local injection	Mice	Ovarian function ↑, fibroblast growth factor 2 ↑	62
	Endometrial disorders	Severe AS	Deliver through the cervix to the fundus of the uterus	Human	Endometrial thickness ↑	70
		Mechanical injured-induced intrauterine adhesion	Local injection	Rat	Pregnancy rate ↑	77

UC-MSCs	Ovarian dysfunction	CTX-induced POF	Tail vein injection	Mice	Weight of the ovaries ↑, estradiol ↑,	80
		Paclitaxel-induced POF	Local injection	Rat	Follicle-stimulating hormone ↓, estradiol ↑, ovarian function ↑	81
		Perimenopausal ovary	Tail vein injection	Rat	Estradiol ↑, follicle-stimulating hormone ↓, follicle number ↑	85
		Busulfan CTX-induced premature ovarian insufficiency	Local injection	Mice	Fertility ↑, ovarian functions ↑	77
	Endometrial disorders	Uterine niche	Local intramuscular injection	Human	Uterine scar reconstruction ↑, uterine niche incidence ↓	91
		95% ethanol-induced endometrial injury	Tail vein injection	Rat	Fertility ↑, endometrial fibrosis ↓, angiogenesis ↑	87

CTX: cyclophosphamide; TG: tripterygium glycosides; ↑: increase; ↓: decrease.
